# Our Book Table

**Published:** 1885-08

**Authors:** 


### OUR BOOK TABLE.

A Guide to the Diseases of Children. By James Frederick Goodhart, M. D., F. R. C. P., Assistant Physician to Guy's Hospital, and Lecturer on Pathology in its Medical School. Physician to the Evelina Hospital for Sick Children. Revised and edited by Louis Starr, M. D., Clinical Professor of Diseases of Children in the Hospital of the University of Pennsylvania; Physician to the Children's Hospital, Philadelphia. With formulæ. Philadelphia: P. Blakiston, Son & Co., 1885.

The medical press is constantly offering to the profession new books upon almost every conceivable topic connected with medicine. Of these, too large a proportion, like Pindar's razors, are made to sell. There is no special reason for their issue, aside from the desire of pecuniary profit, and when this end is attained they quietly drop into a merited oblivion. Publishers contract to deliver books as illuminating gas is laid on—by the volume—and tomes are ground out like sausages, by machinery. Amid all this dreary waste of literary truck, it is gratifying occasionally to meet with a book for the existence of which there is a reasonable excuse. That the subject matter of the one under consideration is of the utmost importance, all will agree. It only remains to inquire if it be treated in a rational and instructive manner.

For a treatise upon the diseases of children, it must be evident that the points of observation should be mainly clinical. Philosophical disquisitions, and turgid, formal dissertations, are quite out of place. And herein lies the chief merit of Goodhart. While embracing the whole subject, and covering all the ground, there is no appearance of a desire to make a special display of learning, and certainly there is no "padding." The work is a plain, common-sense treatise upon the diseases of childhood, and it may be implicitly followed as a safe and sure guide.

MARYLAND MEDICAL JOURNAL.

This is one of the best of the Weekly Medical Journals of the country. We always open it in the confident anticipation of finding something that shall interest and instruct, and that expectancy is never disappointed. To say that it is well edited implies very much more than the average reader can comprehend. It is only an editor who can appreciate the immense amount of labor involved in licking into shape the crude and carelessly written communications that make up the larger part of the contents of a journal. There is many an educated, cultured man, who cannot command language that shall read smoothly. There are a thousand inelegances of speech, colloquialisms, redundancies of expression and transpositions, to say nothing of gross grammatical errors and rhetorical inaccuracies, that the editor must correct, even in the manuscript of the best of writers. So much, too, depends upon the punctuation, the paragraphing, and the judicious use of capitals and different fonts of type, that a well edited journal becomes, to the initiated, a real work of art. A good editor is an expert in the use of language, and his journal gives evidence of the fact in every page. The casual reader, whose attention is directed to the euphonious language of an article and the elegance of its diction, perhaps little imagines that it is to the "editing" that his admiration is due. Especially in the reports of society meetings is this the case. What would speakers say if editors printed their speeches precisely as they are delivered, or even as they reach the editorial table in the reports? It is to this careful and able editing that journals like the one whose name is at the head of this article, and the *Weekly Medical Review*, owe much of their charm. The latter, in spite of the rather frequent change of editors, has always retained its literary merit. One of these journals is eastern, having its home in Baltimore; the other, western, hailing from St. Louis. They differ in characteristics, but are both alike in presenting points of excellence that amply account for their extended circulation.

THE PHYSICIAN HIMSELF, *and what he should add to his scientific acquirements in order to secure success.* By D. W. CATHELL, M. D., late Professor of Pathology in the College of Physicians and Surgeons, of Baltimore. Fourth Edition. Baltimore: Cushings & Bailey, 1885.

The popularity of this volume may be judged from the fact that this is the fourth edition that has been demanded. There are a thousand things that are requisite to the successful physician. Many a man of good general scholarship and great professional knowledge has seen himself distanced in the race of life simply because he lacked the practical knowledge, the worldly wisdom, the professional tact, the ethical information which it is the object of this book to inculcate. In reading it, one is led to wonder from what source Dr. Cathell draws all his lore. From the lessons of his own life? Has "long experience made him sage"? Is he to be pictured to the minds-eye with shining pate and flowing silver beard? Or are the shrewd aphorisms and the sapient instructions due to his observations of the habits and methods of eminently successful physicians? We incline to the opinion that he is a Lacon, and that his sententious advice and precepts are due to his own perspicuity and intuition. From whatever source they may be derived, there is no question about their value and importance. It is the duty of every physician who would succeed, to make a study of the art as well as of the science of medicine, and we could commend him to no better authority in the former than this book. When the whole of medical practice shall be taught in any school, it will, no doubt, be added to its list of text-books.

THE OLEATES. *An Investigation into their Nature and Action.* By JNO. V. SHOEMAKER, M. D. Philadelphia: F. A. Davis Att'y, Publisher, 1885.

The practice of inunction, or rubbing the body with oils and fatty substances, is of extreme antiquity. It has formed a part of the religious ceremonies of nearly all nations, and we know that from time immemorial the priests of different forms of religion have used their knowledge of remedies to supplement their sacerdotal influence. That there is medicinal virtue in particular oils has always been a matter of belief, and modern chemical science has separated the fatty acids and radical bases, and demonstrated their nature and action. It has even gone further, and united the fats and oils with different drugs, and thereby produced a new class of remedies, which have proved very effective in the control of certain diseases, particularly affections of the skin. The work under notice is devoted to a consideration of this class of medicaments. Dr. Shoemaker has been largely instrumental in introducing them to the profession, and his book should be read by every one engaged in the healing art.

HAY FEVER, *and its Successful Treatment by Superficial Organic Alteration of the Nasal Mucus Membrane.* By CHARLES E. SAJOUS, M. D. Philadelphia: F. A. Davis Att'y, Publisher, 1885.

Many theories have been advanced to account for the origin of this peculiar and distressing complaint. Helmholtz believed it to be due to the presence of micro-organisms. Dr. Beard to a neurasthenic condition. Drs. Daly and Roe to a local chronic diseased condition. Dr. Harrison Allen to an obstruction, either permanent or temporary, of the passages. Dr. Mackenzie called it a periodic coryza. But the general impression has been that the condition is due to a special irritation induced by the pollen of plants. The author of this work believes it due to a variety of causes, which produce a changed and abnormal condition of the nasal and palatal tissues, and the treatment recommended is founded upon these etiological views. Hay fever sufferers, as well as physicians, may be greatly instructed and benefited by a perusal of this volume.

BERLIN AS A MEDICAL CENTRE. *A guide for American Practitioners and Students.* By HORATIO R. BIGELOW, M. D., Sandy Hook, Conn. New England Publishing Co., 1885.

So many practitioners and students are continually going abroad for the purpose of pursuing their professional studies, that a book of this kind will prove extremely useful. It gives complete information concerning all that it is requisite to know; routes of travel, expenses, means of living, telegraph and postal guides, a map of Berlin, with all necessary information concerning one's every day life there. It also gives an account of the advantages offered for professional study, of matriculation, lectures, clinics and hospitals. In fact, it is a complete *vade mecum* of its subject. Every intending tourist would do well to read it, but for the medical student who proposes to go to Berlin, it will prove essential.

Transactions of the New York Odontological Society for 1884. Philadelphia: The S. S. White Dental Manufacturing Company.

The papers and discussions of this society always attract much attention, for it is anticipated that, like a court of last resort, it will pronounce the final decision of the profession. It has in its membership so many men of exceptional intelligence, and comprises so much of technical information, that, no matter what may be the subject matter under consideration, something interesting and instructive is certain to be said. The volume for 1884 fully sustains the high reputation of the society, and makes a book well worthy of careful study. The imprint of its publishers is a sufficient guaranty for its typography and finish.

"Dental Caries — A Critical Summary." By Henry Sewill, M. R. C. S., L. D. S., England. A Review by F. Searle, D. D. S., Springfield, Mass.

Mr. Sewill is a dogmatic and positive writer, who speaks authoritatively from premises founded upon the observations of others. Without the technical knowledge that should give license to speak magisterially, he assumes to sit in judgment upon those whose work he has never personally verified or disproved. It is little wonder, then, that so logical a writer as Dr. Searle should pierce the joints of his harness in more than one place, and expose the weakness of declarations unsustained by actual demonstration. For every reader of Mr. Sewill's "Critical Summary," we would unhesitatingly prescribe the proper antidote — Dr. Searle's Review.

Manipulation of the Microscope. By Edward Bausch. Rochester, N. Y., 1885. Price, 50 cents.

The author of this little work is the well known mechanical optician of the Bausch and Lamb Optical Co. There are very few men who have so thoroughly mastered the mechanical branches of optical science, while in practical microscopy he possesses an enviable reputation. The book is an unpretentious one, and is principally devoted to the exposition of microscopical technics, but what it has attempted is exceedingly well done. As a book of instruction for microscopical beginners, it is not exceeded in directness and simplicity and perspicuity by any of the numerous works of the kind that have been issued.

THE AMERICAN ADVERTISER REPORTER. Nos. 234 and 235 Broadway, New York.

This is a weekly journal, issued for the benefit of publishers, giving the standing resources and credit-rating of general advertisers. It is a mercantile agency report of a special class, and as such is invaluable to publishers who do a general advertising business. $3.00 per year.

DISINFECTION AND DISINFECTANTS.

Preliminary report made by the Committee on Disinfectants of the American Public Health Association.

*Open Letter from* DR. EDWARD W. JENKS *to* DR. N. S. DAVIS, *Editor of the Journal of the American Medical Association.*

*Does Tobacco produce Amblyopia?* By W. FRANKLIN COLEMAN, M. D., M. R C. S., Baltimore, Md.

*Souvenir of the New York Odontological Society.* Annual Dinner 1885.

*Third Annual Report of the Illinois State Board of Dental Examiners.*

*Rules and Preliminary Organization of the Ninth Session of the International Medical Congress,* to be held in Washington in 1887.

*Programme of the Proceedings of the Thirty-sixth Annual Session of the American Medical Association,* held at New Orleans, 1885.

*Address delivered before the American Academy of Science at the Seventeenth Annual Meeting, held in Boston, November 5th,* 1884. By EDWARD A. HARRIS, D. D. S.

*Surgical Notes from the Case-book of a General Practitioner.* By WILLIAM C. WILE, M. D. Reprint from the *New England Medical Monthly.*

*Reports of the application of Hydrochlorate of Cocaine in Ophthalmology, Otology, Laryngology, Gynæcology, Genito-Urinary, Nasal, Dental, and General Surgery.* Compiled from Medical Literature. Parke, Davis & Co., Detroit and New York.

